# Combating Disease Mongering: Daunting but Nonetheless Essential

**DOI:** 10.1371/journal.pmed.0030146

**Published:** 2006-04-11

**Authors:** Iona Heath

## Abstract

The way forward, argues Heath, relies on our capacity to rediscover courage and stoicism and to realign the relationship between economic, political, and professional interests.


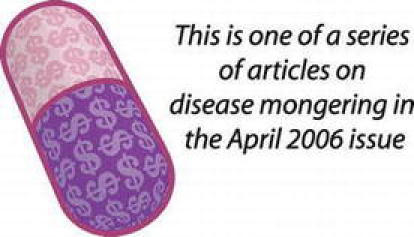


The challenge of combating the current epidemic of disease mongering is daunting, and anyone looking for ready solutions should read no further. Those seeking a way forward find themselves ranged against powerful economic, political, and professional interests. There is an apparently limitless amount of money to be made from marketing pharmaceutical remedies for diseases and even more from remedies to reduce risk factors for disease. An emphasis on the treatment of disease minimises political responsibility for those fundamental causes of disease that are located within the structure of society, and substantial and lucrative professional careers have been built on the endless pursuit of new diseases or risk factors for disease.

More fundamentally, disease mongering exploits the deepest atavistic fears of suffering and death. Throughout history, humanity has kept such fears at bay by accepting burdens and sacrifices in the present in the hope of future salvation. In earlier times, the mediator was religion and the salvation was to come after death. Now, for those without religious belief, death has become more final, and salvation must be sought before death in an ever-expanding longevity. An adequate response to the false hopes raised by disease mongering will demand, from those in positions of power and influence, an ability to acknowledge, accommodate, and move beyond these profound existential fears [ 1]. Such ability is rare.

The way forward will rely on a capacity to rediscover courage and stoicism as both private and civic virtues, alongside seeking a radical realignment of the relationship between economic, political, and professional interests. Doctors and biomedical scientists, in particular, have a responsibility not only to put their own house in order but to provide much better advice to politicians and to the public, both as patients and as citizens.

## The Challenge to Professionals

The first step has to be a genuine disentanglement of the medical profession from the pharmaceutical industry—there really is no such thing as a free lunch [ 2]. The pharmaceutical industry spends millions of dollars supporting the “education” of doctors because it is in its economic interest to do so ( Figure 1). If prescribing activities and industry profits were not affected by this support, it would not be offered. Doctors can only provide appropriately independent and authentic advice to patients and, indeed, to politicians if they are seen to be completely independent of other powerful interests. Politicians genuinely interested in the welfare of patients and the health of citizens should actively promote such independence [ 4].

**Figure 1:**
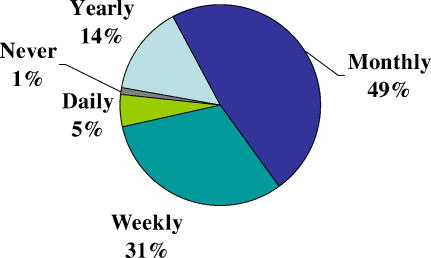
Percentage of Doctors That Use Information Provided by Drug Company Representatives in Their Clinical Practice Data derived from [ 3] (Image: Adapted from a slide presentation created by No Free Lunch, http://www.nofreelunch.org/downloads/Nofreelunch%20Presentation.ppt)

Disease mongering exploits the deepest atavistic fears of suffering and death.

Beyond this, there is a need for better science that has the integrity to demand more explicit acknowledgment of the limits of medical knowledge, less extrapolation beyond research findings, and much more responsible use of statistics, so that the true extent of the benefits and harms of proposed treatments can be properly understood.

The rhetoric surrounding disease mongering suggests that it will promote health, but the effect is in fact the opposite. Much disease mongering relies on the pathologising of normal biological or social variation and on the portrayal of the presence of risk factors for disease as a disease state in itself. When pharmaceuticals are used to treat risk factors, the vicious circle is completed because “anyone who takes medicines is by definition a patient” [ 5].

Most variables are distributed across a continuum, but despite this, the medical tradition has been to dichotomise the continuum into normal and abnormal [ 6]. Within a continuum, there can never be a clear boundary, so the definition of disease is inevitably both arbitrary and fluid. It is in the interests of pharmaceutical companies to extend the range of the abnormal so that the market for treatments is proportionately enlarged. We have seen this process operating, for example, in the continual lowering of thresholds for treatment of blood pressure and lipids—the most recent guidelines from the European Society of Cardiology can be used to identify 76% of the total adult population of a county of Norway as being at “increased risk” [ 7].

We need to reverse this situation so that instead of defining an arbitrary threshold of abnormality, governments would make a judgement about the appropriate level of investment in preventive technologies for currently healthy populations, and set the thresholds for intervention accordingly. For example, a government might decide to treat the 10% of the population most at risk of ischaemic heart disease, and could then calculate the thresholds of blood pressure and cholesterol, which would identify this most vulnerable 10% of the population. Clearly, these thresholds would be much higher than those recommended today.

## Preventing Disease through Population-Based Measures

The seeds of the current situation were already present 21 years ago, when Geoffrey Rose wrote his seminal paper entitled “Sick individuals and sick populations” [ 4]. In this paper, he showed that risk factors for health are almost always distributed across a bell curve and argued that more could be achieved by attempting to shift the whole of the bell curve than by targeting those at highest risk ( Figure 2). His rationale was that the large number of people at low risk may give rise to more cases of disease than the small number who are at high risk. There has been general acceptance of this argument, but Rose's own warnings seem to have been lost.

**Figure 2:**
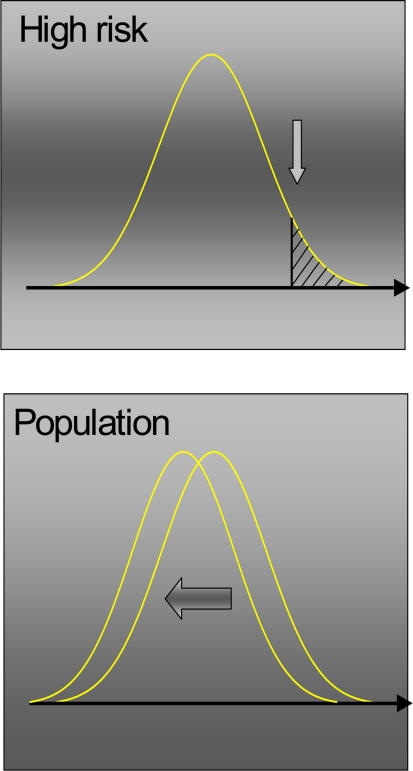
Risk Factors for Health Are Almost Always Distributed across a Bell Curve Geoffrey Rose argued that more could be achieved by attempting to shift the whole of the bell curve (the “population approach” to prevention) than by targeting those at highest risk (the “high-risk” approach). (Image: Adapted from a figure by John Emberson from [ 8])

Rose was careful to list the *disadvantages* of attempting to shift the whole bell curve, which included (1) a “worrisome” benefit–risk ratio (there is only a small possibility of benefit for any one individual, but everyone is exposed to the intervention and thereby to any adverse effects, such as medicalisation, anxiety, and side effects of treatments) and (2) poor motivation on the part of individual patients, each of whom had a very small chance of benefit. These predicted disadvantages have become more and more apparent, so there has been a systematic attempt to improve motivation through the explicit use of fear, which in itself erodes and undermines health. “If you don't take more exercise, improve your diet or take this medication, you actively put yourself at risk of an untimely death.”

Rose was also very careful to distinguish between two approaches to shifting the bell curve. The first restores biological normality by preventing exposure to hazards such as tobacco smoke or industrial air pollution. The second approach is to interpose some new, supposedly protective intervention, but this is much less robust because it leaves the underlying causes intact. The current use of pharmaceuticals for public health policy falls into this category. As more and more risk factors are identified, closely followed by a pharmaceutical treatment for each, the ambition to shift the whole bell curve legitimises the wholesale drug treatment of healthy populations at vast expense and with huge pharmaceutical profits. There is a clear need to reiterate Rose's distinction and prioritise the reduction of exposure to biological hazards above the application of pharmaceutical prophylaxis.

Current trends raise the prospect of exponential spending on preventive pharmaceuticals, justified by potential long-term benefits to an unidentifiable, but statistically significant, number of people in the population. When doctors treat patients with diseases, progress can be assessed and the outcome is measurable. This means that if the patient responds to treatment, it can be continued; if not, the treatment can be stopped. When doctors treat people who are merely at risk of disease, the outcome is probabilistic, so whether disease is prevented or was never going to develop, the treatment continues indefinitely [ 9].

Shifting the bell curve through population-based interventions aimed at protecting health is part of a long and magnificent tradition which began when John Snow capped the Broad Street pump; shifting the bell curve through the mass pharmaceutical treatment of individuals turns out to be something quite different. Further, where individualised solutions become prevalent, societal, population-based interventions tend to fall away, and the result is worsening health inequalities.

The medical profession needs to do much more to define sensible limits to medical intervention. There is a clear and urgent need for more research into the psychological impact and the wider health consequences of being labelled “at risk” [ 10]. Doctors, and society as a whole, need to stop confusing health with happiness [ 11]. This confusion is at the root of much of the medicalisation of normal human variation that we are witnessing. Male pattern baldness and shyness, to take just two examples, are not diseases but normal parts of the range of human experience. We are witnessing diagnostic drift in a whole range of conditions, from depression [ 12] to hypertension [ 13], with pressure for more and more people to be included within the range of abnormal and offered treatment. The justification for these treatments is often based on short-term studies, which are then extrapolated over much longer time periods. There is insufficient recognition of the fact that the less the need for treatment, the higher the number needed to treat for given outcomes and the higher the risk to patients, since the rate of adverse effects remains constant.

## The Challenge to Politicians

Politicians are charged with overseeing the organisation of society for the benefit of all. A major political achievement of Western societies, with the very notable exception of the United States, has been the provision of universal health-care systems available and accessible to all. There is now a pressing need for politicians to recognise the threat to these systems, and to the social solidarity that they embody, posed by exponential increases in pharmaceutical expenditure. No universal health-care system funded by taxation can pay for the pharmaceutical treatment of all risks to health. There are very difficult decisions to be made, but politicians must balance the wish to support a vibrant and innovative pharmaceutical industry—and the undoubted economic, employment, and therapeutic benefits that drug companies bring—against the increasing capacity of this industry to bankrupt universal health-care systems.

Part of the rationale for expenditure on the treatment of health risks is that it will reduce health costs in the long run, but such arguments do not stand up to close scrutiny. The costs of health care are highest during the year before death, regardless of the age at which death occurs. Everyone must die and be cared for while dying, and no amount of preventive pharmaceuticals can reduce the cost of providing this crucial end-of-life care [ 14]. The aim of preventive health care should be the reduction of untimely deaths in relatively young people, but the fear of accusations of ageism in health care means that doctors are encouraged to go on prescribing preventive pharmaceuticals to people well into their late eighties and nineties [ 15].

In any system of health care predicated on social solidarity, the rights of individuals to treatment have to be balanced against the duties of citizens to provide the appropriate level of funding. Citizens agree to pay tax for the care of those who are sick, with the understanding that they, too, will be cared for should they, in turn, become sick. It remains unclear how far this pact of social solidarity extends to paying for the treatment of risk factors and marginal “diseases”, where the benefits in terms of reducing suffering are much less clear. Publicly funded preventive treatment of risk factors for those who have already exceeded the average life expectancy seems particularly hard to justify.

Socioeconomic deprivation has been described as a “fundamental cause” of disease, which works through a multiplicity of risk factors and pathophysiological pathways to produce multiple disease states [ 16]. Even if one of these pathways is interrupted by the application of a preventive technology, an association between a fundamental cause and disease will reappear in a different form. The closer to the individual the intervention is situated, the less likely the improvement in health status is to be maintained.

In mental health problems, we see this process operating when people are helped to cope with poor housing and lack of rewarding employment through the provision of counselling, rather than better social conditions. Population-based interventions favour the poor because such interventions are applied universally and the poor are the most at-risk; individually based interventions favour the rich because they are more likely to make use of what is offered. For this reason, population approaches to tackling the fundamental causes of socioeconomic deprivation must remain the most effective way of tackling health inequalities [ 17]. However, there is also a continuing role for individually based treatment of those at the highest risk of particular diseases, but this must be effectively targeted if inequalities are not to be exacerbated.

Age is another fundamental cause of disease, less remediable than poverty but again generating multiple risk factors and multiple disease outcomes. All clinicians are familiar with the processes by which treating one disease in a frail, older person will often mean that symptoms reappear through another pathway. Authentic health care for the old and frail has much more to do with helping to preserve their dignity, treating them with affection, and supporting their continued involvement in social activities, rather than the pursuit of ever-more elusive cures. Politicians have a responsibility, alongside doctors and many others, to make such care available, and this in itself will be an important part of the way forward.

The huge amount of money that can be made from preventive technologies has diminished the economic importance of treatment technologies, particularly for those illnesses that primarily affect poorer people in poorer countries [ 18]. This has meant a shift of attention from the sick to the well and from the poor to the rich [ 19]. This effect of global markets requires a response in the form of an assertion of global human solidarity. Health inequalities matter globally as well as locally. A way forward might be through taxation or other means, to make the sale of preventive technologies in countries with above-average expectation of life conditional on the availability of treatment for those diseases that cause the most catastrophic shortening of life in poor countries.

## Conclusion

Human societies are riven by the effects of greed and fear. The rise of preventive health technologies has opened up a new arena of human greed, which responds to an enduring fear. The greed is for ever-greater longevity; the fear is that of dying. The irony and the tragedy is that the greed inflates the fear and poisons the present in the name of a better, or at least a longer, future. Ultimately, the only way of combating disease mongering is to value the manner of our living above the timing of our dying.
